# Open Long-Tailed Multimodal 3D Model Classification Based on Sample-Enhanced Category-Space Learning

**DOI:** 10.3390/jimaging12080370

**Published:** 2026-08-10

**Authors:** Yuansa Wang, Xueyao Gao, Chunxiang Zhang, Yongzeng Xue

**Affiliations:** 1 School of Computer Science and Technology, Harbin University of Science and Technology, Harbin 150080, China; 2210402075@stu.hrbust.edu.cn (Y.W.); zhangchunxiang@hrbust.edu.cn (C.Z.); 2 Faculty of Computing, Harbin Institute of Technology, Harbin 150001, China; xue@hit.edu.cn

**Keywords:** computer vision, multimodal 3D model classification, feature fusion, open long-tailed learning, incremental learning, mamba, hard sample

## Abstract

With the rapid development of three-dimensional (3D) sensing technologies, multimodal 3D model classification has achieved significant progress. However, most existing methods are developed under closed and balanced assumptions, which limits their applicability to open long-tailed scenarios with scarce tail classes, ambiguous hard samples, and continuously emerging categories. In this work, we propose sample-enhanced category-space learning (SE-CSL) for open long-tailed multimodal 3D model classification. The proposed method first uses dual-branch modality encoders to extract point-cloud structural representations and multi-view semantic representations. Mamba is then introduced to model global dependencies across heterogeneous modalities and generate a unified global category representation. To improve the robustness of category representation, we design a category-space learning strategy that jointly integrates long-tailed learning, few-shot representation stabilization, and hard-sample enhancement. A long-tail balanced loss, a few-shot stabilization loss, and a hard-sample boundary loss are further developed to optimize intra-class compactness, inter-class separability, and boundary discrimination. To handle continually emerging classes, we introduce an incremental category-space expansion mechanism that distinguishes new classes, preserves old-class information, and supports unified classification of old and new categories. Extensive experiments on ModelNet40 and ShapeNet55 demonstrate the effectiveness and robustness of SE-CSL.

## 1. Introduction

Robust recognition remains challenging in open long-tailed 3D classification because tail classes contain few samples, their decision boundaries are ambiguous, and a single modality often provides insufficient evidence. Advances in artificial intelligence [1], 3D sensing [2], and digital modeling [3] have rapidly increased both the volume of available 3D data and the complexity of real-world classification scenarios. Compared with two-dimensional (2D) images, 3D data encode richer geometric structures, spatial topology, and shape details, which are valuable for object understanding and semantic analysis. Recent multimodal 3D classification methods improve representation learning through cross-modal pretraining, adaptive view modeling, and unified point-view encoding [4,5,6,7,8,9,10]. Long-tailed and few-shot learning methods further address class frequency bias, difficult samples, and scarce-category representations [11,12,13,14,15]. However, these problems are generally investigated under separate learning objectives, while their joint treatment in a progressively expanding multimodal category space remains limited. When few-shot categories are introduced sequentially, the imbalance between previously learned and newly added categories may further affect category representation and classification stability [16,17]. Consequently, existing category spaces are often imbalanced, unstable, and insufficiently extensible for accurate multimodal 3D classification under open long-tailed conditions.

In the proposed method, openness is reflected by the progressive expansion of the category space, where future categories are introduced with only a few labeled samples. The long-tailed setting describes the imbalanced training-sample distribution among the base classes, which are divided into head, medium, and tail classes according to their sample sizes. The class-incremental setting introduces previously unseen categories sequentially over multiple sessions. After each session, the model is evaluated on all categories observed up to the current stage. Therefore, long-tailed learning addresses the sample imbalance in the initial category space, and class-incremental learning supports its progressive expansion.

To address these challenges, SE-CSL is proposed for open long-tailed multimodal 3D model classification. It constructs a balanced and extensible category space from complementary point-cloud and multi-view representations.

The main contributions of this paper are as follows:

(1) Sample-enhanced category-space learning framework. We propose SE-CSL, a unified framework for open long-tailed multimodal 3D model classification. Instead of treating multimodal fusion, long-tailed recognition, hard-sample discrimination, and incremental learning as isolated procedures, SE-CSL organizes them within a category-space learning paradigm. This design promotes stable, balanced, and extensible category representations and improves classification robustness in challenging open long-tailed scenarios.
(2) Dual-modality feature extraction with global dependency modeling. A dual-modality feature extractor jointly exploits point-cloud geometry and multi-view appearance semantics. The point-cloud encoder captures spatial structures and local geometric patterns, whereas the multi-view encoder extracts semantic cues from rendered views. Mamba models long-range contextual dependencies across heterogeneous features, thereby strengthening cross-modal interaction and producing a more complete global representation.
(3) Category-space learning under long-tailed and hard-sample conditions. The category-space learning strategy jointly integrates long-tailed rebalancing, few-shot stabilization, and hard-sample enhancement. It reduces head-class dominance, stabilizes scarce-class representations, and sharpens ambiguous decision boundaries. A unified optimization objective further improves intra-class compactness, inter-class separability, and discrimination near category boundaries.
(4) Incremental category-space expansion. An incremental expansion mechanism supports the continual introduction of new classes. It updates new-class representations while preserving old-class information in the learned category space, which enables unified classification of old and new categories without severe degradation of previously acquired knowledge.

The remainder of this paper is organized as follows. Section 2 reviews single-modal and multimodal 3D classification methods, together with related learning strategies. Section 3 describes the proposed method. Section 4 presents the experimental results, ablation studies, and visual analyses. Section 5 concludes the paper and discusses future research directions.

## 2. Related Work

This section reviews the studies most closely related to the proposed method, including single-modal approaches, multimodal approaches, and related learning strategies.

### 2.1. Single-Modal Methods

Early point-cloud methods establish direct learning from unordered point sets. PointNet uses a symmetric aggregation function to produce global representations [18], whereas PointNet++ partitions points into local neighborhoods and extracts hierarchical multi-scale features [19]. Subsequent work extends this paradigm toward relation-aware and attention-based architectures. Point Geometry Transformation (PointGT) applies local geometric transformations to strengthen structural descriptions [20]. The Graph Transformer Network (GTNet) integrates graph construction with Transformer blocks to connect local neighborhoods and global context [21]. Point Transformer V3 simplifies the Point Transformer architecture and improves scalability and efficiency [22]. PointMamba replaces quadratic self-attention with a state-space model for efficient global context modeling [23]. PointGeo enhances local and global feature extraction through geometric transformations [24], and The Point Agent Transformer Network (PAT-Net) introduces point agents and dual-frequency feature extraction to strengthen contextual dependency modeling [25].

Early multi-view methods establish image-based 3D representation learning. The multi-view convolutional neural network (MVCNN) renders each 3D model from multiple viewpoints and aggregates the resulting features through view pooling [26]. RotationNet jointly predicts object categories and poses, which enables latent viewpoint relations to be learned without explicit pose supervision [27]. More recent methods advance from static view aggregation to deep cross-view interaction. iMVS, which integrates multi-view information on multiple scales, models multi-scale view features using view and channel Transformers [28]. Walk in Views combines a path-aggregation graph network with a path Transformer to propagate information across views and construct global shape descriptors [29]. The view-set Transformer (VSFormer) treats the rendered images as a flexible view set and explicitly models pairwise and higher-order correlations [30]. The Patch–View–Shape Progressive Interaction Transformer (PVSTrans) progressively connects patch-, view-, and shape-level information to capture local regions, cross-view relations, and global semantics [31]. Subsequent studies place greater emphasis on adaptive view selection. Multi-View Fusion PointCLIP (MVF-PointCLIP) suppresses noisy views and assigns larger weights to informative views in zero-shot classification [32], whereas a prototype-based fine-grained method extends multi-view recognition toward interpretable and prototype-driven discrimination [33].

Multi-view methods capture texture and projection-based semantics, whereas point-cloud methods directly preserve spatial structure and geometric topology. Both, however, rely on a single modality. Discrete rendered views only approximate the underlying geometry, which limits discrimination of fine shape details, local structures, and occluded objects. Point clouds, in contrast, provide limited appearance and cross-view information. The joint use of point-cloud and multi-view data is therefore required to construct a more complete 3D representation.

### 2.2. Multimodal Methods

PointCLIP, a Contrastive Language–Image Pre-training (CLIP)-based point-cloud understanding method, projects point clouds into multi-view depth maps and aligns 3D categories with CLIP text features, establishing a practical route from vision–language pretraining to 3D classification [34]. PointMCD, a multi-view cross-modal distillation method, transfers visual knowledge from multiple views to point encoders and narrows the gap between 2D appearance and 3D geometry, thereby formalizing cross-modal distillation for 3D shape recognition [35]. More recent CLIP-based and large-scale pretraining methods further extend 3D representation learning. Unified Language–Image–Point representation learning (ULIP) aligns point-cloud representations with a shared image–text space through tri-modal pretraining [36], whereas CLIP2Point transfers CLIP knowledge through image-depth contrastive pretraining and efficient adaptation [37]. OpenShape scales text–image–point-cloud alignment to large 3D collections for open-world shape understanding [38]. Uni3D constructs a scalable 3D foundation model by aligning point-cloud features with pretrained image–text representations [39], and ULIP-2 improves scalability through automatically generated language descriptions and large-scale tri-modal learning [40]. MM-Point, a multi-view information-enhanced multimodal self-supervised method, combines point clouds and multi-view images with intra- and inter-modal similarity objectives for self-supervised multimodal pretraining [41]. Differentiable rendering-based multi-view Image–Language Fusion (DILF) integrates adaptive views with image–language fusion for zero-shot 3D classification [42], and GeoZe transfers vision–language features from views to points while enforcing geometric consistency [43]. PointTFA, a data-efficient training-free 3D adaptation method, adapts unified language–image–point models without additional parameter updates and uses representative support samples for data-efficient classification [44]. OpenView unifies point clouds and multi-view images through a cross-modal fusion encoder [45]. ViewCloud represents multi-view projection cues in a point-cloud-like structure to bridge 2D images and 3D points efficiently [46]. MVF-PointCLIP suppresses noisy views and reweights informative views for zero-shot classification [], whereas Fusion of Language and Point Clouds for Classification (FLPC) directly aligns language and point-cloud features without image intermediaries [47].

These multimodal methods improve 3D representation through cross-modal alignment, adaptive view modeling, and unified encoding. Their principal objectives include self-supervised pretraining, zero-shot transfer, data-efficient adaptation, and multimodal fusion. However, they do not jointly address long-tailed base-class learning, hard-sample optimization, and few-shot class-incremental expansion within a unified setting. This limitation motivates the construction of a category space that supports multimodal representation, imbalanced learning, and progressive expansion.

### 2.3. Related Modeling Methods

Class-balanced loss reweights classes according to their effective numbers of samples and provides an early solution to long-tailed recognition [48]. Balanced Meta-Softmax corrects the bias of standard Softmax under skewed class distributions [49], whereas Focal Loss reduces the contribution of easy samples and emphasizes difficult examples [50]. Prototypical Networks represent each class by a prototype and establish a simple metric-based framework for few-shot learning [51]. For few-shot point-cloud classification, Cross-Instance Adaptation addresses large intra-class variation and small inter-class differences [52]. More recently, Point Cloud Mamba replaces quadratic attention with a state-space model for efficient global point-cloud modeling [53].

Multi-scale fusion and attention mechanisms have also been extensively investigated in light-field image processing. Cross-Stage Partial Darknet-53 (CSPDarknet53) and bidirectional feature pyramid networks are combined to aggregate hierarchical features and recover information across different spatial scales. Multi-scale receptive fields and feature pyramid refinement are further used to capture local structures and long-range contextual information. Dense attention, channel attention, and progressive refinement strategies are introduced to emphasize informative regions and suppress interference caused by occlusion. In addition, nested U^2^-Net structures are employed to strengthen hierarchical feature extraction, and Swin Transformer-based de-occlusion networks capture long-range dependencies among light-field views. Multi-stage attention-guided U^2^-Net++ architectures further integrate spatial pyramid representation, feature pyramid fusion, and progressive reconstruction to recover occluded structures and improve feature stability [54,55,56,57,58,59,60,61]. Although these studies primarily focus on light-field image reconstruction or salient object detection, their concepts of multi-scale aggregation, long-range dependency modeling, and selective feature enhancement provide valuable insights for complex visual representation learning. Class-balanced loss and Balanced Meta-Softmax reduce the prediction bias caused by imbalanced class frequencies, and Focal Loss emphasizes difficult samples during optimization. Prototype-based methods further improve category representation under few-shot supervision. Class-incremental studies also indicate that the sequential introduction of new classes may increase the imbalance between old and new categories and affect the retention of previously learned knowledge. These studies provide important foundations for the proposed method, while long-tailed learning, few-shot representation, hard-sample discrimination, and multimodal category expansion are generally investigated under separate learning settings.

Table 1 compares representative methods from multimodal 3D classification, open-world 3D representation, long-tailed recognition, few-shot learning, and class-incremental learning. The distinction of SE-CSL lies in the coordinated learning and expansion of a balanced and extensible multimodal category space.

## 3. Method

**Figure 1:**
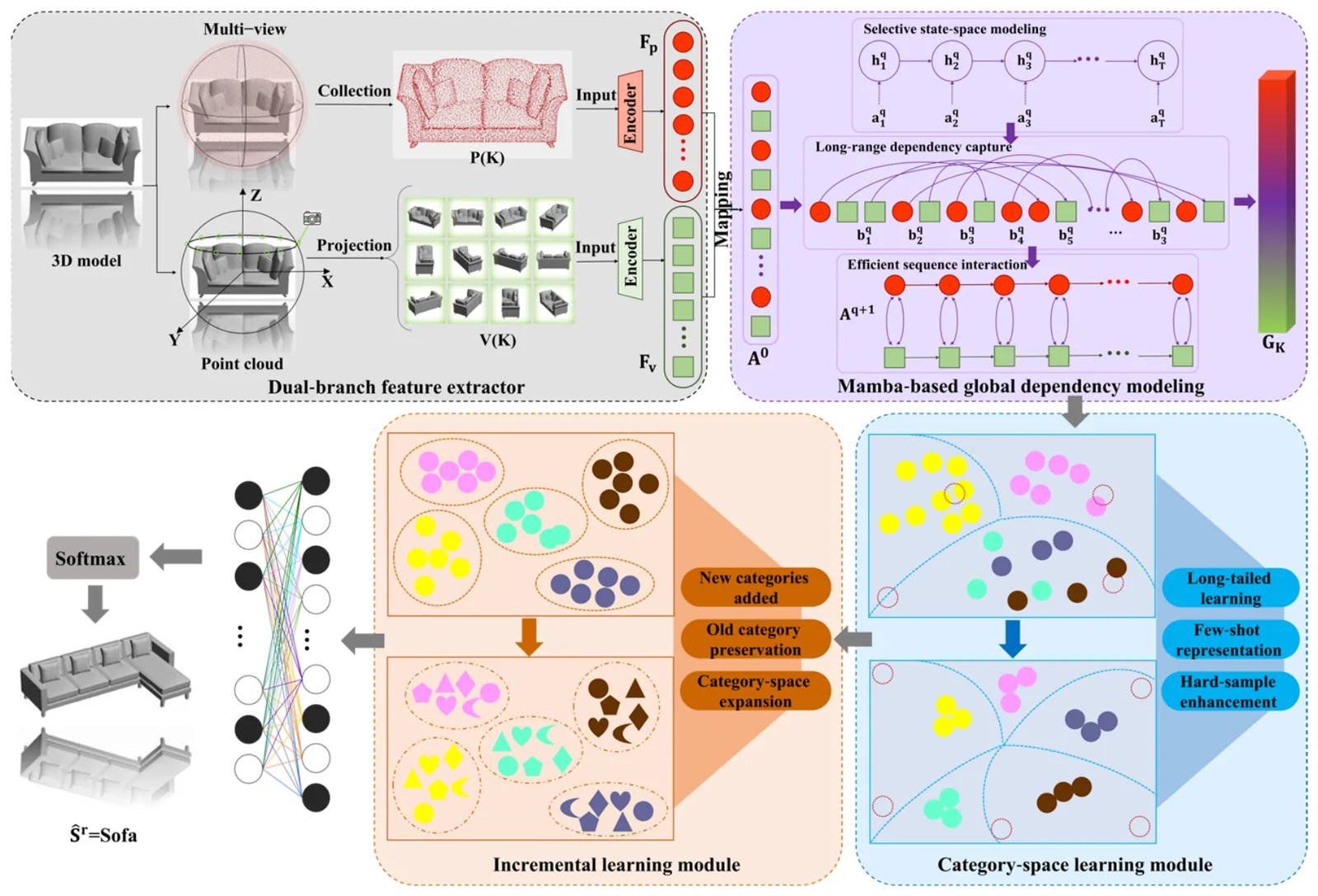
Implementation framework of the proposed SE-CSL method. Each 3D model is represented by a point cloud containing N = 1024 points and M = 12 rendered views. The point-cloud encoder contains four EdgeConv layers with output dimensions of 64, 64, 128, and 256, and the number of nearest neighbors is set to k = 20. The concatenated point-cloud feature is projected into a 512-dimensional embedding. ResNet50 with [3, 4, 6, 3] bottleneck blocks is used as the multi-view encoder, and each 2048-dimensional view feature is projected into the same 512-dimensional space. The resulting multimodal sequence is processed using Mamba with State Dim = 16, Kern = 6, and Expan = 2. The unified representation is subsequently optimized by category-space learning and incremental category-space expansion for classification over all observed classes. Arrows indicate the forward flow of data and features, and different background colors distinguish the major processing stages of SE-CSL.

Figure 1 presents the overall architecture of the proposed method.

### 3.1. Dual-Branch Feature Extractor

A 3D model is denoted as K, and the total number of categories C. As shown in Figure 1, the category set is $S=\{S_{c}{\}}_{c=1}^{C}$, where $S_{c}$ denotes the c-th semantic category. Point clouds and multi-view images are jointly used to represent the geometric structure and appearance of each 3D model. Specifically, a point-cloud set $P(K)=\{p_{n}|n=1,2,\ldots ,1024\}$ is obtained from K through uniform surface sampling, where $p_{n}\in R^{3}$ denotes the n-th sampled point on the model surface and encodes local geometry and spatial shape. A multi-view set $V(K)=\{v_{m}|m=1,2,\ldots ,12\}$ is generated through fixed-view rendering, where $v_{m}$ denotes the m-th rendered view and captures contour and appearance information from a specific observation angle. Based on these two modalities, a point-cloud encoder and a multi-view encoder are constructed to extract modality-specific features, where $\theta_{p}$ and $\theta_{v}$ denote the parameters of the two encoders. Specifically, Dynamic Graph Convolutional Neural Network (DGCNN) is adopted as the point-cloud backbone. It contains four edge-convolution (EdgeConv) layers with output dimensions of 64, 64, 128, and 256, respectively, and the number of nearest neighbors is set to 20. The features generated by the four EdgeConv layers are concatenated and projected into a 512-dimensional point-cloud embedding. ResNet50 is adopted as the multi-view backbone, and its four residual stages contain 3, 4, 6, and 3 bottleneck blocks, respectively. The final classification layer is removed, and global average pooling is applied to obtain a 2048-dimensional feature for each rendered view. A linear projection layer then maps each view feature into the same 512-dimensional embedding space as the point-cloud feature. ResNet50 is initialized using ImageNet-1K pretrained weights. DGCNN is trained from scratch, and its convolutional and fully connected layers are initialized using Xavier uniform initialization. The newly introduced projection layers, Mamba layers, and classification layers are also initialized using Xavier uniform initialization, and their bias terms are initialized to zero. For all batch-normalization layers, the scale and bias parameters are initialized to one and zero, respectively. All encoder and projection parameters are optimized end-to-end on the target task. Therefore, the point-cloud feature $F^{p}$ and each view feature in $F^{v}$ are represented in a common 512-dimensional embedding space before being sent to the subsequent Mamba-based global dependency modeling process.

### 3.2. Mamba−Based Global Dependency Modeling

Mamba uses selective state-space modeling to propagate long-range context with linear sequence complexity. It progressively accumulates information from point-cloud and multi-view tokens. As shown in Figure 1, the heterogeneous inputs $F_{p}$ and $F_{v}$ are mapped into a unified multimodal sequence $A_{0}$. This mapping is defined in Formula (1).

(1) $$A_{0}=\left[\Phi_{p}\left(F_{p}\right)+e^{p};\Phi_{v}\left(F_{v}\right)+e^{v}\right]+e^{pos},A_{0}\in R^{T\times d}$$

where $\Phi_{p}(\cdot )$ and $\Phi_{v}(\cdot )$ denote the point-cloud and multi-view mapping functions, respectively. They project $F_{p}$ and $F_{v}$ into the common Mamba hidden dimension d. $e^{p}$ and $e^{v}$ denote the corresponding modality embeddings, which retain token-specific modality information. $e^{pos}$ denotes the positional embedding, which provides positional information within the unified sequence. The semicolon denotes concatenation along the token dimension, d denotes the token feature dimension, and T denotes the total number of concatenated multimodal tokens. $A_{0}$ is then processed by the stacked Mamba layers. For the q-th Mamba layer, the input sequence is $A_{q}={\left[a_{1}^{q},a_{2}^{q},\ldots a_{T}^{q}\right]}^{⊺}\in R^{T\times d}$ and contains T multimodal tokens. State recursion propagates long-range context while storing information accumulated from preceding tokens. The recursive state sequence of the *q*-th Mamba layer is defined as $H_{q}={\left[h_{1}^{q},h_{2}^{q},\ldots h_{T}^{q}\right]}^{⊺}\in R^{T\times d}$. $H_{q}$ is transformed into the context-enhanced response sequence $B_{q}={\left[b_{1}^{q},b_{2}^{q},\ldots b_{T}^{q}\right]}^{⊺}\in R^{T\times d}$, which encodes information associated with the current token. A gated residual connection then updates the input $A_{q}$ using $B_{q}$ before it is passed to the next layer. The update is defined in Formula (2).

(2) $$A_{q+1}=A_{q}+\Theta_{out}^{q}(B_{q}⊙\sigma (\Theta_{gates}^{q}(A_{q})))$$

where $A_{q+1}$ denotes the output sequence of the q-th Mamba layer, q∈{0,1,…,Q−1}, and Q denotes the total number of stacked Mamba layers. $\Theta_{out}^{q}\left(\cdot \right)$ denotes the output mapping function in the q-th Mamba layer, and $\Theta_{gates}^{q}\left(\cdot \right)$ denotes the interaction gating function. σ(⋅) denotes the Sigmoid activation function, and ⊙ denotes element-wise multiplication. The output tokens of the final layer are globally averaged and normalized to obtain the unified representation $G_{K}$ for the 3D model, as defined in Formula (3).

(3) $$G_{K}=LN(\frac{1}{T}\sum_{t=1}^{T}a_{t}^{Q})$$

where $a_{t}^{Q}$ denotes the t-th output token of the final Mamba layer, and LN(⋅) denotes layer normalization. $G_{K}$ contains point-cloud geometric information, multi-view semantic information, and long-range dependencies between heterogeneous modalities. This representation therefore captures global dependencies between point-cloud and multi-view features.

### 3.3. Category-Space Learning Module

**Figure 2:**
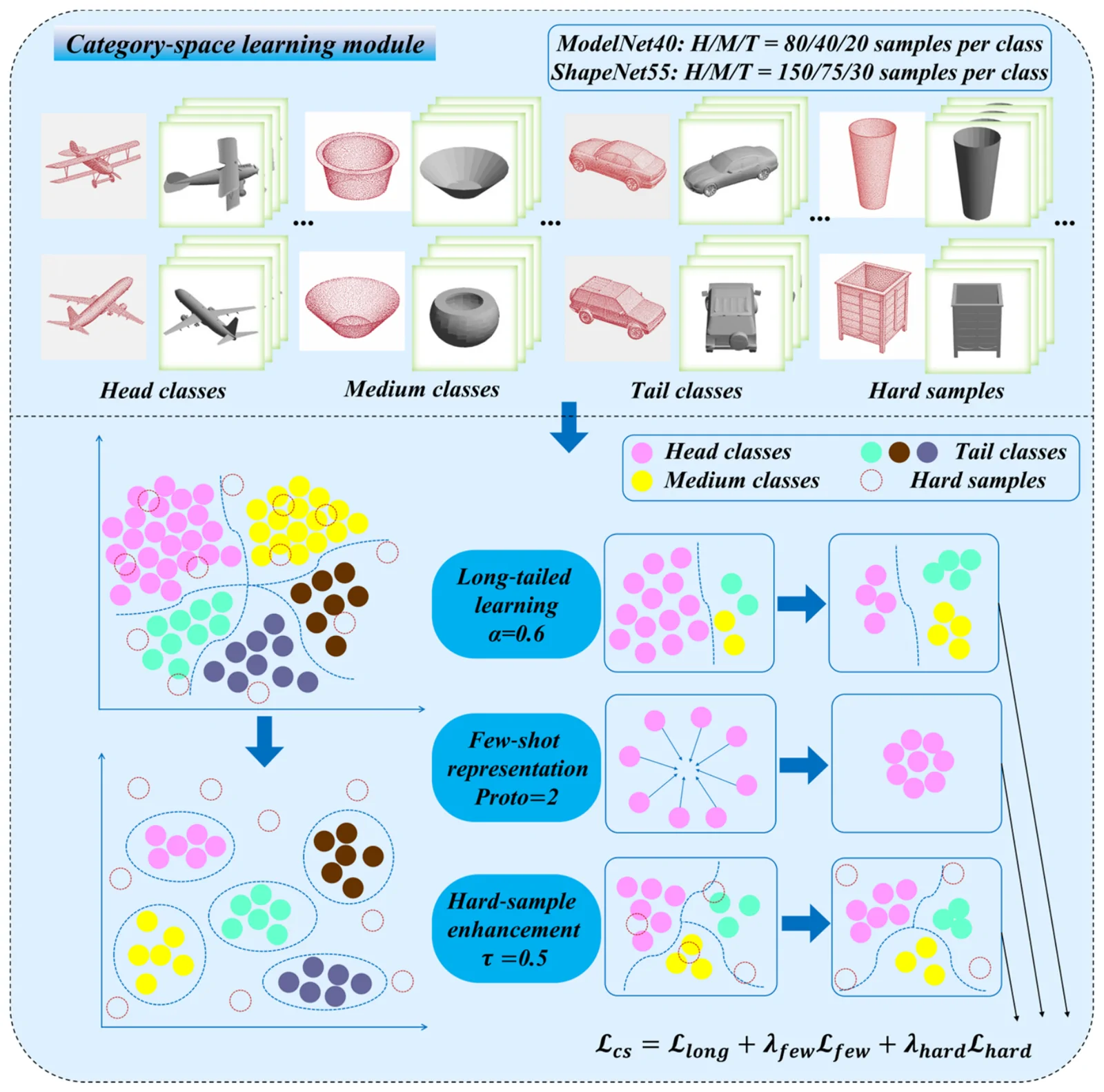
Schematic diagram of the category-space learning module.

Representations of different 3D categories often overlap in feature space. Under a long-tailed distribution, head classes contain sufficient samples and typically form stable category representations, whereas medium and tail classes contain fewer samples and are more susceptible to boundary compression by head classes. As shown in Figure 2, the category-space learning module addresses this problem through long-tailed rebalancing, few-shot stabilization, and hard-sample enhancement.

Long-tailed learning reduces the class bias induced by imbalanced sample frequencies. Head-class regions are typically more stable and can dominate optimization, thereby compressing the decision regions of medium and tail classes. To increase the influence of underrepresented classes, a class-balance coefficient is derived from class frequency and applied to the classification constraint. The category-space transformation function ϕ(⋅) is used to map the global multimodal representation $G_{K}$ into a common category space. It is a learnable transformation shared by the base-training and incremental learning stages, and its parameters are optimized jointly with the feature encoders. Therefore, representations generated at different training stages remain in the same category space and can be directly compared with the corresponding category centers. $L_{long}$ constrains the category-space representation of the 3D model K. The representation is encouraged to approach its ground-truth center while remaining separated from competing centers. The long-tailed learning constraint is defined in Formula (4).

(4) $$L_{long}=-\kappa_{\chi (K)}\log\frac{exp(sim\left(\varphi \left(G_{K}\right),\pi_{\chi \left(K\right)}\right))}{\sum_{c=1}^{C}exp(sim\left(\varphi \left(G_{K}\right),\pi_{c}\right))}$$

where $\kappa_{\chi \left(K\right)}$ denotes the class-balance coefficient assigned to the ground-truth category of K. It is a fixed class-dependent coefficient determined according to the predefined head, medium, and tail partition. Classes within the same group use the same coefficient, and medium and tail classes receive progressively stronger weights than head classes. The balancing strength is controlled by Alpha, and the coefficient remains unchanged during training. $\pi_{c}$ denotes the category center of category $S_{c}$, and χ(K) denotes the ground-truth category index of K. Each category center is obtained from the mean category-space representation of the available training samples belonging to that category and is refreshed as the feature representations are optimized. C denotes the number of currently considered categories. sim(⋅,⋅) denotes cosine similarity between two category-space representations, where a larger value indicates a smaller angular difference and a stronger semantic correspondence. During training, the influence of head classes is reduced, and the separability of long-tailed classes is improved.

Tail classes and newly introduced classes often exhibit dispersed feature distributions and unstable category centers. Few-shot stabilization therefore imposes a spatial constraint that draws samples toward their corresponding category centers. $L_{few}$ denotes the few-shot stabilization term, and the corresponding training set T is used to define the constraint in Formula (5).

(5) $$L_{few}=\frac{1}{\left|T\right|}\sum_{K\in T}\beta_{\chi (K)}{∥\varphi \left(G_{K}\right)-\pi_{\chi \left(K\right)}∥}_{2}^{2}$$

where $\beta_{\chi \left(K\right)}$ denotes the few-shot stabilization coefficient of the ground-truth class. Rather than being computed from the exact number of samples, it is assigned according to the predefined head, medium, and tail groups. Classes within the same group share a common value, whereas tail and newly introduced few-shot classes receive larger coefficients and therefore stronger intra-class constraints. As multimodal features from the same class are aggregated in category space, the category center becomes more stable and intra-class compactness is improved.

Classes with similar global contours are difficult to separate near category boundaries. Hard-sample enhancement therefore measures the similarity margin between the ground-truth class center and the nearest competing center. The resulting boundary constraints are defined in Formulas (6) and (7).

(6) $$L_{hard}=\frac{1}{\left|T\right|}\sum_{K\in T}I(\delta_{K}<\tau ){\left(\tau -\delta_{K}\right)}^{2}$$

(7) $$\delta_{K}=sim\left(\varphi \left(G_{K}\right),\pi_{\chi \left(K\right)}\right)-\underset{c\neq \chi \left(K\right)}{\max}\left(\varphi \left(G_{K}\right),\pi_{c}\right)$$

where $L_{hard}$ denotes the hard-sample enhancement term. $\delta_{K}$ denotes the category margin of K. τ denotes the hard-sample threshold, and I(⋅) denotes the indicator function. When $\delta_{K}$ is small, the sample lies close to a decision boundary and is easily confused with adjacent classes. Such a sample is treated as a hard example and receives an additional boundary constraint. The total category-space learning loss is then defined in Formula (8):

(8) $$L_{CSL}=L_{long}+\lambda_{few}L_{few}+\lambda_{hard}L_{hard}$$

where $\lambda_{few}$ and $\lambda_{hard}$ denote the weight coefficients of the few-shot stabilization loss and the hard-sample boundary loss, respectively. The three loss terms provide complementary constraints for category-space optimization. $L_{long}$ increases the optimization contribution of tail classes through class-dependent reweighting, which reduces head-class dominance. $L_{few}$ minimizes the distance between scarce-class samples and their category centers, which reduces intra-class dispersion and stabilizes category representations. $L_{hard}$ penalizes samples with insufficient similarity margins, which enlarges the separation between the ground-truth category and the nearest competing category. Therefore, their joint optimization improves class balance, intra-class compactness, and inter-class boundary discrimination under open long-tailed conditions.

### 3.4. Incremental Learning Module

**Figure 3:**
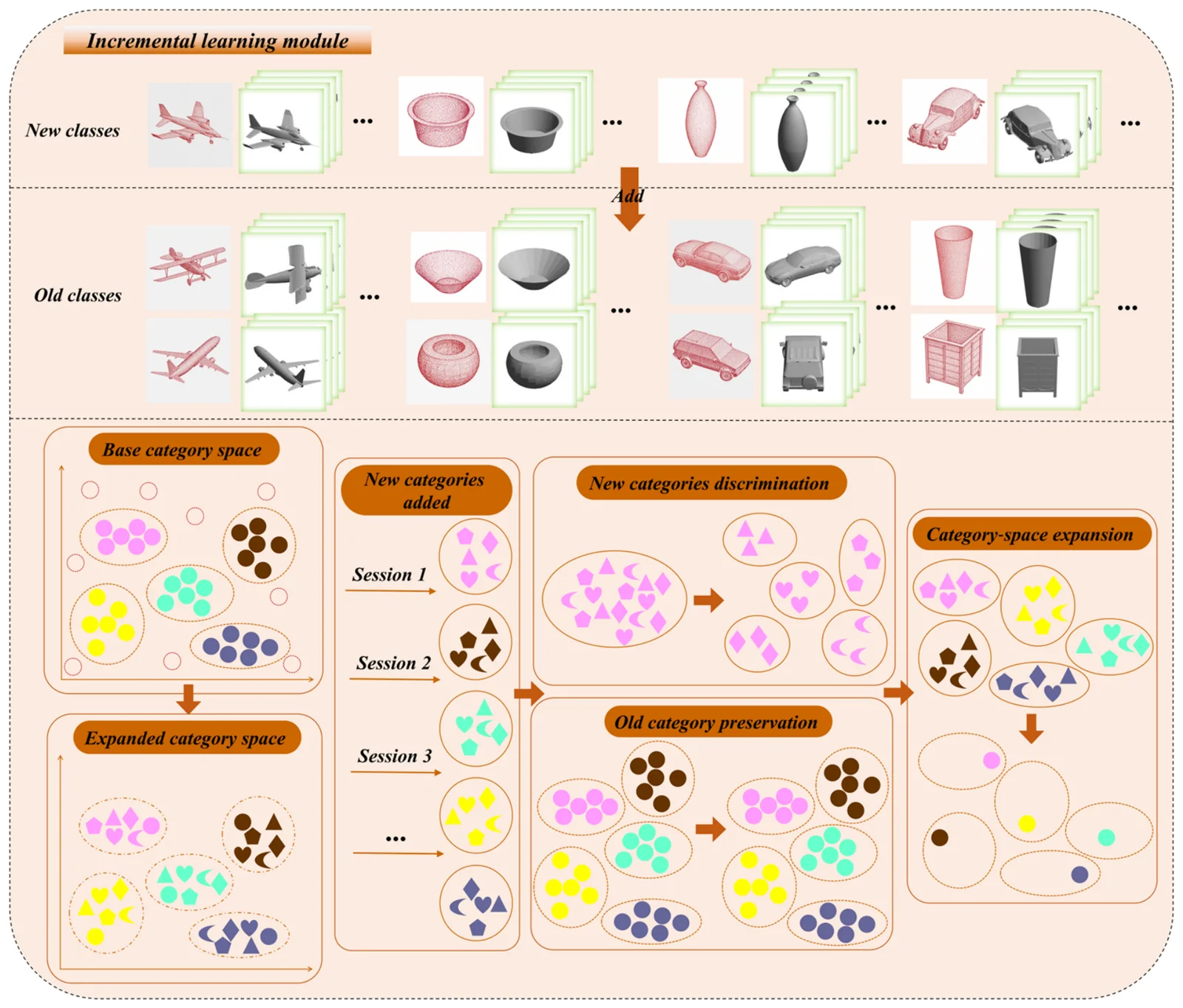
Schematic diagram of the incremental learning module. Different colors distinguish semantic categories, and different marker shapes indicate categories introduced during different incremental sessions. Arrows indicate the direction of category-space expansion and knowledge transfer.

The incremental learning module enables the category space to expand under open-world conditions. New categories are introduced with only a few labeled samples, and the discriminative structure of previously learned categories must be retained. As shown in Figure 3, category-space expansion comprises new-class addition, new-class discrimination, old-class preservation, and unified classification. Newly introduced classes are therefore learned together with all previously observed classes.

At the *r*-th incremental stage, the set of previously learned classes is $O_{r}$, and the set of newly introduced classes is $N_{r}$. Classification is performed over all classes observed by this stage, denoted by $U_{r}=O_{r}\cup N_{r}$. The shared category-space transformation φ(⋅) projects $G_{K}$ into the learned category space, producing the feature set $F_{new}^{r}$ for new-class samples at the r-th stage. The transformed features of the newly introduced classes are defined in Formula (9).

(9) $$F_{new}^{r}=\{\varphi (G_{K})|S_{\chi \left(K\right)}\in N_{r}\}$$

For each class $S_{c}$ introduced at the r-th stage, the class center $\pi_{c}^{r}$ is computed using Formula (10).

(10) $$\pi_{c}^{r}=\frac{1}{n_{c}^{r}}K\in D_{c}^{r}\varphi \left(G_{K}\right),S_{c}\in N_{r}$$

where $n_{c}^{r}$ denotes the number of available samples of category $S_{c}$ at the r-th incremental stage, and $D_{c}^{r}$ denotes the corresponding sample set. The center of each base category is initialized as the mean category-space representation of all available training samples belonging to that category. At the r-th incremental stage, the center of each newly introduced category is initialized by averaging the transformed representations of its few-shot samples according to Formula (10). For an old category, the category center stored after the previous stage is used to initialize its center at the current stage and is retained as the reference center for knowledge preservation. To stabilize the representations of newly introduced categories and restrict the displacement of old-category centers, the incremental learning loss is constructed, as shown in Formula (11).

(11) $$L_{in}^{r}=L_{CSL}^{r}+\lambda \sum_{S_{c}\in O_{r}}{∥\pi_{c}^{r}-\pi_{c}^{r-1}∥}_{2}^{2}$$

where $L_{CSL}^{r}$ denotes the category-space learning loss at the r-th incremental stage. $\pi_{c}^{r}$ and $\pi_{c}^{r-1}$ denote the category centers of old category $S_{c}$ at the current and previous stages, respectively, and λ denotes the weight coefficient of the old-category center preservation term. The category space is thereby expanded to include all observed classes. The final representation $F^{r}$ is passed to a Softmax classifier, where $F_{l}^{r}$ denotes the score for class $S_{l}$. The prediction is defined in Formulas (12) and (13).

(12) $$P_{l}^{r}=\frac{exp(F_{l}^{r})}{\sum_{c=1}^{C^{r}}exp(F_{c}^{r})}$$

(13) $${\hat{S}}^{r}=S_{arg\underset{1\leq l\leq C^{r}}{\max}P_{l}^{r}}$$

where $C^{r}$ denotes the number of all seen categories at the r-th incremental stage, $l\in \left\{1,2,\ldots ,C^{r}\right\}$ denotes the category index, and $P_{l}^{r}$ denotes the predicted probability of category $S_{l}$. ${\hat{S}}^{r}$ denotes the category with the maximum predicted probability among all seen categories $S_{1},S_{2},\ldots ,S_{C^{r}}$.

## 4. Experiments

### 4.1. Datasets

ModelNet40 [50] and ShapeNet55 [51] are used to evaluate the proposed method. ModelNet40 contains 40 object categories and 12,311 3D models, including 9843 training samples and 2468 testing samples. ShapeNet55 contains 55 object categories and 46,513 3D models, including 36,147 training samples and 10,366 testing samples. Occluded-ModelNet40 and Rendered-ModelNet40 are additionally used to assess robustness under degraded observation conditions.

### 4.2. Experimental Setup

All experiments are conducted under the same training and testing environment. SE-CSL is implemented using Python 3.10 and PyTorch 2.1.0 with CUDA 11.8 and NumPy 1.24.4, and is trained on a single NVIDIA GeForce RTX 4090 graphics processing unit (GPU; NVIDIA Corporation, Santa Clara, CA, USA). Adam is used with an initial learning rate of 0.001, and each model is trained for 200 epochs. Inference latency is averaged over 1000 forward passes after 50 warm-up iterations, with GPU synchronization performed immediately before and after timing. Peak GPU memory is measured using torch.cuda.max_memory_allocated (). For reproducibility, the random seeds of Python, NumPy, and PyTorch are fixed. The class partitions and training samples are generated once and retained for all compared methods. Each experiment is repeated independently using seeds 0, 1, and 2, and the mean and standard deviation are reported. For the principal overall accuracy (OA) results, 95% confidence intervals are calculated using Student’s t-distribution. The resulting intervals are [93.69%, 94.63%] for ModelNet40 and [88.42%, 89.82%] for ShapeNet55. Each 3D model is represented by 1024 uniformly sampled points and 12 rendered views captured from fixed viewpoints. FLOPs and parameter counts are measured under this common input setting. OA, mean class accuracy (mAcc), recall, precision, and F1-score are used to evaluate classification performance.

Table 2 compares the proposed protocol with established settings for long-tailed classification, class-incremental learning, few-shot class-incremental learning, open-set recognition, and open-world 3D recognition.

A fixed class partition is used throughout the open long-tailed incremental protocol. Class assignments are defined once and are not resampled across runs. For ModelNet40, 20 base classes are divided into 8 head, 6 medium, and 6 tail classes, with 80, 40, and 20 training samples retained per class, respectively. The remaining 20 classes are divided into four fixed five-way five-shot incremental sessions. For ShapeNet55, 25 base classes are divided into 10 Head (H), 8 Medium (M), and 7 Tail (T) classes, with 150, 75, and 30 training samples retained per class, respectively. The remaining 30 classes are divided into six fixed five-way five-shot sessions. Samples are selected without replacement from the official training splits using a fixed random seed, and the same class assignments and sample indices are used for all methods and repeated runs. The official test splits remain unchanged, and evaluation includes all classes observed up to the current session, as summarized in Table 3.

### 4.3. Comparative Experiment

To evaluate SE-CSL comprehensively, we compare it with conventional multi-view, point-cloud, and multimodal 3D classification methods. Because classification accuracy alone does not fully reflect practical applicability, Floating-point operations (FLOPs) and parameter counts are also reported to quantify computational complexity and model size. The results are presented in Table 4.

As shown in Table 4, SE-CSL achieves the highest OA on ModelNet40 (94.16%), exceeding the strongest listed baseline, ViT-GE, by 0.66 percentage points. It outperforms Pointwise CNN and Gao-PointNet by 7.09 and 5.76 percentage points, respectively, and exceeds the CLIP-based multi-view methods Prompt-Enhanced View Aggregation Network (PEVA-Net) and Multi-View Contrastive Language–Image Pre-training method (MV-CLIP) by 9.68 and 9.72 percentage points. These results indicate that point-cloud geometry and multi-view appearance provide complementary category evidence. On ShapeNet55, SE-CSL achieves an OA of 89.12%, outperforming PEVA-Net and MV-CLIP by 14.47 and 22.95 percentage points, respectively. It also exceeds the point-cloud masked-autoencoder baselines Point Feature Enhancement Masked Autoencoder (Point-FEMAE) and Predicting Centers for Point Masked Autoencoders method (PCP-MAE) by 0.49 and 0.88 percentage points. SE-CSL requires 18.9 G FLOPs and 38.6 M parameters. Although this cost exceeds that of lightweight point-cloud baselines, it remains substantially below that of CLIP-based multi-view methods, which require more than 70 G FLOPs and approximately 150 M parameters. SE-CSL therefore provides a favorable balance between classification accuracy and computational complexity.

Table 5 further compares SE-CSL with recent methods for 3D incremental learning and open-world recognition, and the forgetting metric is also reported to evaluate performance degradation across incremental sessions.

SE-CSL maintains competitive classification performance and a lower forgetting value, which indicates more stable knowledge retention during incremental category-space expansion.

Table 6 summarizes the generalization performance and computational efficiency of SE-CSL on ShapeNet55 and ModelNet40. Recall, precision, F1-score, mAcc, OA, training time, inference latency, peak GPU memory, and scalability jointly characterize classification quality and practical computational cost across datasets of different scales.

On ShapeNet55, SE-CSL achieves an OA of 89.12% and an mAcc of 83.27%; on ModelNet40, it achieves an OA of 94.16% and an mAcc of 91.75% (Table 6). Recall, precision, and F1-score also remain stable on both datasets, which indicates balanced classification performance. Similar inference latency and memory consumption are observed across the two datasets, and category-space expansion introduces only limited additional overhead. These results support the generalization ability, computational feasibility, and scalability of SE-CSL.

**Figure 4:**
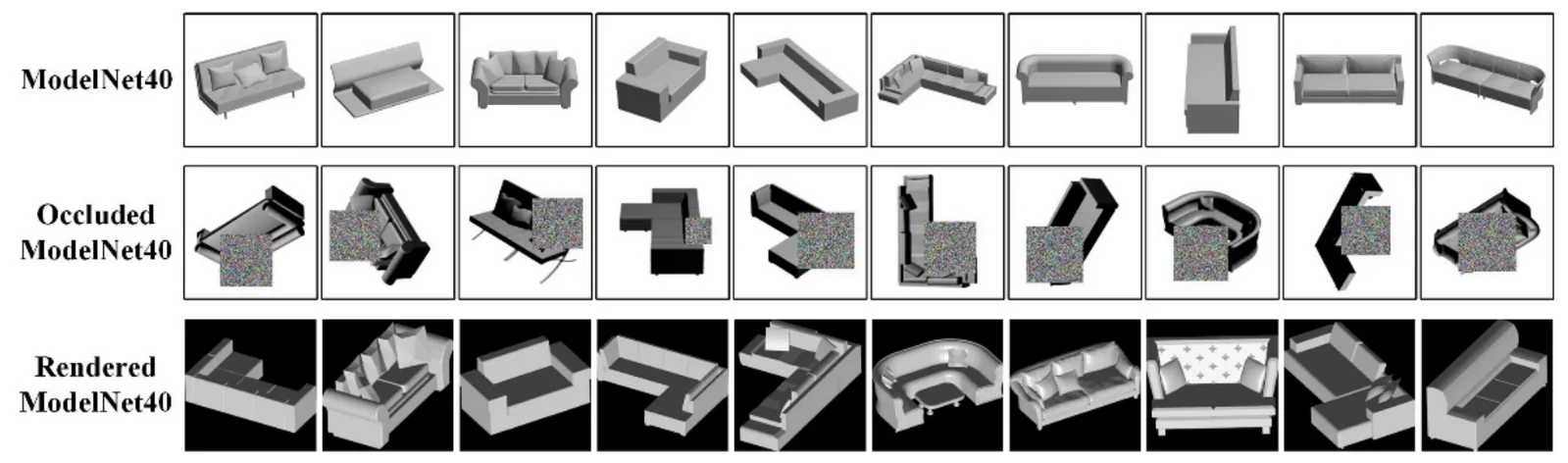
Different ModelNet dataset variants.

To assess robustness under degraded conditions, SE-CSL is evaluated on Occluded-ModelNet40 and Rendered-ModelNet40 alongside representative baselines and four controlled variants: SE-CSL without Mamba, SE-CSL with Transformer layers, SE-CSL without category-space learning (CSL), and SE-CSL without incremental learning (IL) (Table 7). The Transformer variant replaces each Mamba layer with a Transformer encoder layer. For self-attention, sequence interaction and attention-memory complexities are O (T^2^d) and O (T^2^), respectively, whereas selective state-space propagation in Mamba scales linearly with sequence length T. Classification performance and computational cost are therefore compared under identical conditions. As illustrated in Figure 4, Occluded-ModelNet40 introduces occlusions into ModelNet40 samples to simulate incomplete observations, whereas Rendered-ModelNet40 evaluates stability under viewpoint and appearance variations.

SE-CSL achieves OAs of 80.82% on Occluded-ModelNet40 and 88.53% on Rendered-ModelNet40 (Table 7). The moderate decline relative to ModelNet40 indicates that the method remains robust to occlusion and partial observations. Its representations retain both geometric awareness and a degree of view invariance across categories with different structural characteristics. The Transformer variant requires 20.5 G FLOPs and 42.1 M parameters, whereas Mamba-based SE-CSL requires 18.9 G FLOPs and 38.6 M parameters. Their inference latencies on ModelNet40 are 33 and 30 ms, respectively. By avoiding explicit pairwise attention, Mamba achieves a better balance between classification accuracy and computational cost.

### 4.4. Performance Experiment

The effects of the Mamba state dimension (Dim), convolution kernel size (Kern), and expansion factor (Expan) are evaluated through parameter experiments. Dim is set to 8, 16, or 32; Kern is set to 2, 4, or 6; and Expan is set to 1, 2, or 4. Each parameter combination is trained and tested independently, and the corresponding OA values are reported in Table 8.

It is observed that the best performance is achieved when Dim is set to 16, Kern is set to 6, and Expan is set to 2. The highest OA values are 89.12 ± 0.28% on ShapeNet55 and 94.16 ± 0.19% on ModelNet40. Dim = 16 provides sufficient state capacity for global dependency modeling, whereas Kern = 6 supports effective local context interaction and feature propagation. Expan = 2 provides moderate channel expansion without destabilizing feature transformation. These results show that balanced parameter settings improve Mamba-based feature learning and 3D classification performance.

To investigate the key parameters of category-space learning, experiments are conducted on the long-tail balancing parameter Alpha, the global few-shot stabilization factor Proto (ρ), and the hard-sample selection threshold τ. Proto controls the overall strength of the few-shot stabilization loss, whereas the class-specific coefficient $\beta_{\chi \left(K\right)}$ is assigned according to the predefined head, medium, and tail groups. Alpha is set to 0.3, 0.6, or 0.9; Proto is set to 1, 2, or 3; and τ is set to 0.35, 0.5, or 0.65. The results are reported in Table 9. A small *τ* value selects only highly ambiguous samples and may provide insufficient boundary supervision, whereas a large value includes relatively easy samples and may impose excessive boundary constraints. The best performance on both datasets is obtained when *τ* = 0.5. This threshold is therefore used in subsequent experiments to balance hard-sample selectivity and boundary coverage.

It is observed that the best performance is achieved when Alpha is set to 0.6, Proto is set to 2, and *τ* is set to 0.5. The highest OA values reported in Table 9 are 89.12 ± 0.28% on ShapeNet55 and 94.16 ± 0.19% on ModelNet40. A moderate long-tail balancing strength reduces category bias, whereas an appropriate few-shot stabilization weight preserves discriminative structures for scarce classes.

Class-incremental performance is evaluated on ModelNet40 and ShapeNet55, as reported in Table 10. For ModelNet40, 20 of the 40 categories form the base phase, and five new categories are added in each of four sessions (S1–S4), increasing the number of observed categories from 20 to 40. For ShapeNet55, 25 of the 55 categories form the base phase, and five new categories are introduced in each of six sessions (S1–S6), increasing the number of observed categories from 25 to 55. At every stage, the model classifies all categories observed up to that point.

In the base phase, SE-CSL achieves 95.52 ± 0.15% mAcc and 96.29 ± 0.56% OA on ModelNet40, and 88.52 ± 0.51% mAcc and 92.65 ± 0.63% OA on ShapeNet55 (Table 10). Performance gradually decreases as new categories are introduced. Nevertheless, the final ModelNet40 session retains 91.75 ± 0.35% mAcc and 94.16 ± 0.19% OA, whereas the final ShapeNet55 session retains 83.27 ± 0.63% mAcc and 89.12 ± 0.28% OA. These results indicate that SE-CSL learns new-class representations while preserving substantial old-class knowledge. Although the introduction of new geometric structures and appearance patterns increases confusion among old and new classes, the method remains robust throughout long-tailed class-incremental learning.

### 4.5. Ablation Experiment

Ablation experiments are conducted to quantify the contribution of each component (Table 11). Component (a) denotes the point-cloud modality, (b) the multi-view modality, (c) Mamba-based dependency modeling, (d) category-space learning, and (e) incremental category-space expansion.

Each component of SE-CSL contributes to classification performance (Table 11). Combining point-cloud and multi-view inputs consistently improves mAcc and OA relative to either single-modality setting, which confirms the complementarity of geometric and appearance information. Mamba provides a gain by modeling global dependencies in the multimodal sequence and strengthening cross-modal interaction. Category-space learning improves intra-class compactness and inter-class separability, whereas incremental expansion preserves old-class information when new categories are introduced. The relatively small standard deviations indicate stable training, and most component-wise gains exceed the corresponding variation across repeated runs.

The contributions of the three category-space losses are further evaluated: the long-tail balancing loss *L*_long_, the few-shot stabilization loss *L*_few_, and the hard-sample boundary loss *L*_hard_. Their individual and joint effects on ShapeNet55 and ModelNet40 are reported in Table 12.

Compared with the setting without the three category-space losses, the individual use of $L_{long}$ improves mAcc and OA by 3.12 and 1.46 percentage points on ShapeNet55, respectively. On ModelNet40, the corresponding improvements are 1.75 and 1.08 percentage points. These results indicate that long-tail balancing effectively reduces the prediction bias caused by uneven category distributions. When only $L_{few}$ is used, mAcc and OA increase by 1.54 and 0.44 percentage points on ShapeNet55, and by 2.41 and 0.81 percentage points on ModelNet40. This result shows that few-shot stabilization improves the representation consistency of categories with limited training samples. The individual use of $L_{hard}$ improves mAcc and OA by 2.99 and 1.39 percentage points on ShapeNet55, and by 2.00 and 0.49 percentage points on ModelNet40. This confirms that hard-sample supervision strengthens the discrimination of samples near category boundaries. When all three losses are combined, SE-CSL achieves the best performance: 83.27 ± 0.63% mAcc and 89.12 ± 0.28% OA on ShapeNet55, and 91.75 ± 0.35% mAcc and 94.16 ± 0.19% OA on ModelNet40. The long-tail balancing, few-shot stabilization, and hard-sample boundary losses therefore provide complementary supervision. Their joint optimization improves distributional balance, intra-class compactness, and inter-class separation under open long-tailed conditions.

**Figure 5:**
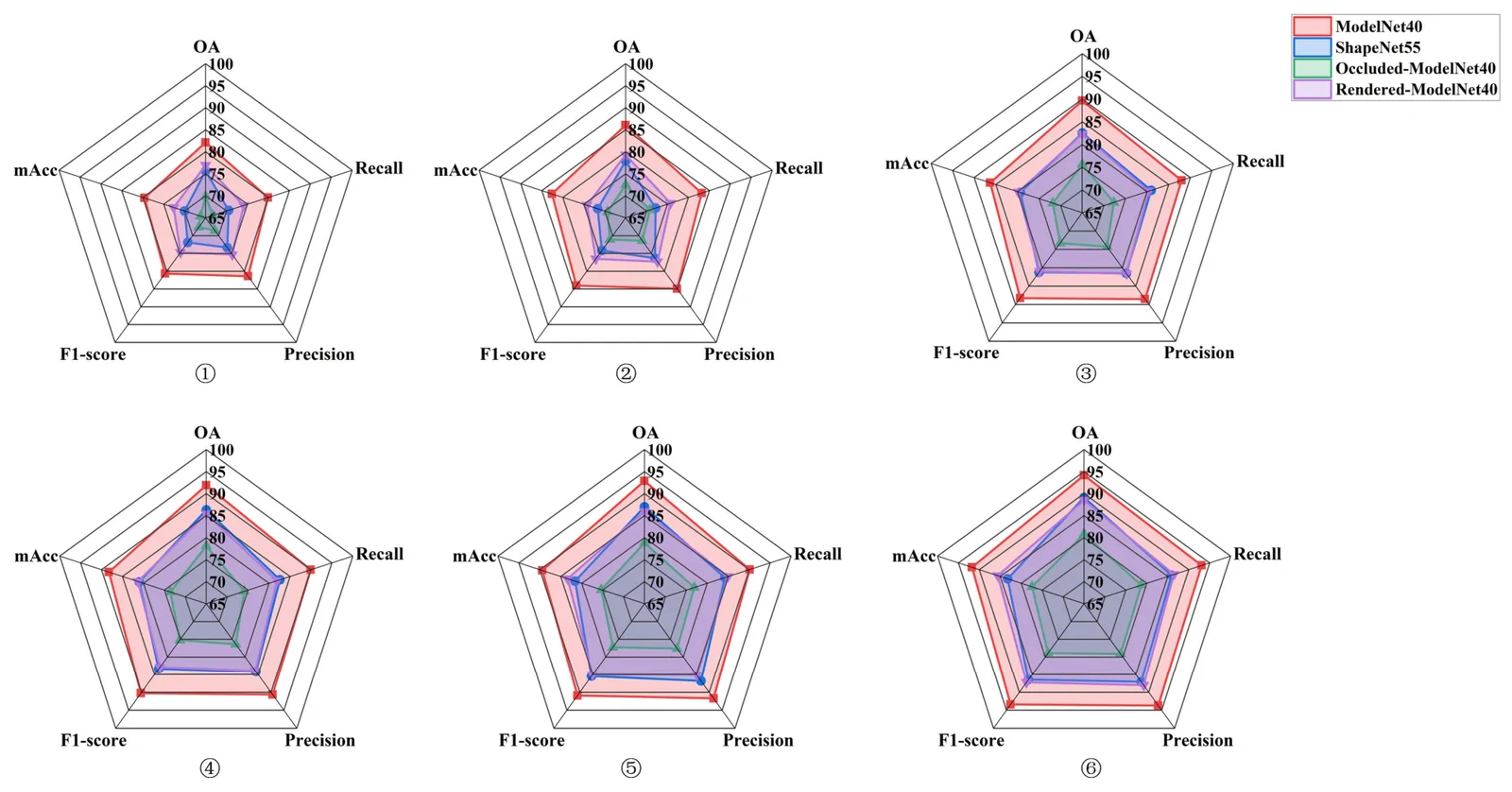
Radar chart visualization of ablation results. Configurations ①–⑥ correspond to the ablation settings in Table 11: ① point-cloud modality only; ② multi-view modality only; ③ point-cloud and multi-view modalities; ④ configuration ③ with Mamba-based dependency modeling; ⑤ configuration ④ with category-space learning; and ⑥ the complete SE-CSL model with incremental category-space expansion.

Figure 5 summarizes the ablation results across multiple metrics. OA, recall, precision, F1-score, and mAcc are compared on ShapeNet55, ModelNet40, Occluded-ModelNet40, and Rendered-ModelNet40, which provides an intuitive view of the overall differences among component combinations.

### 4.6. Visualization and Failure-Case Analysis

**Figure 6:**
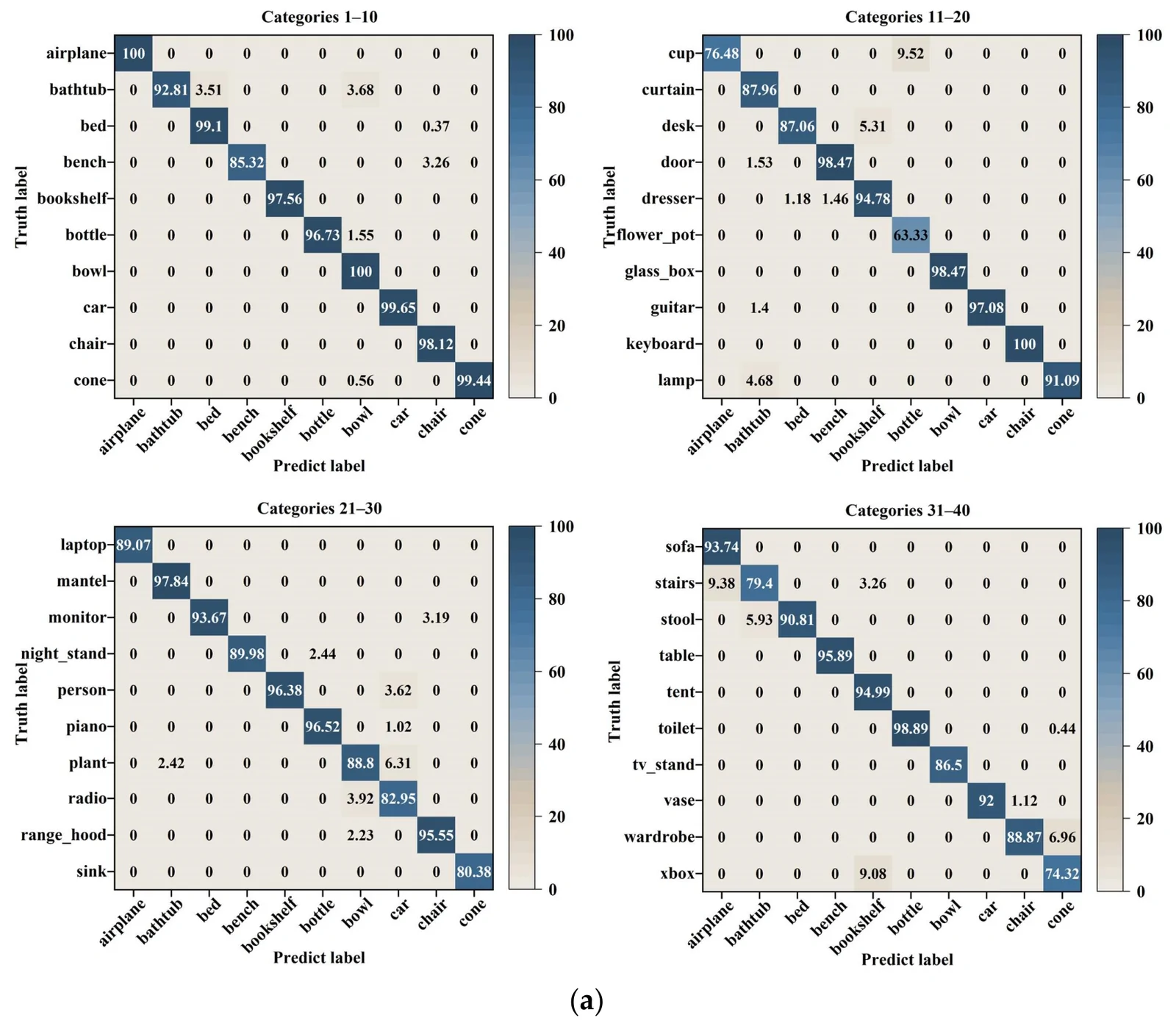

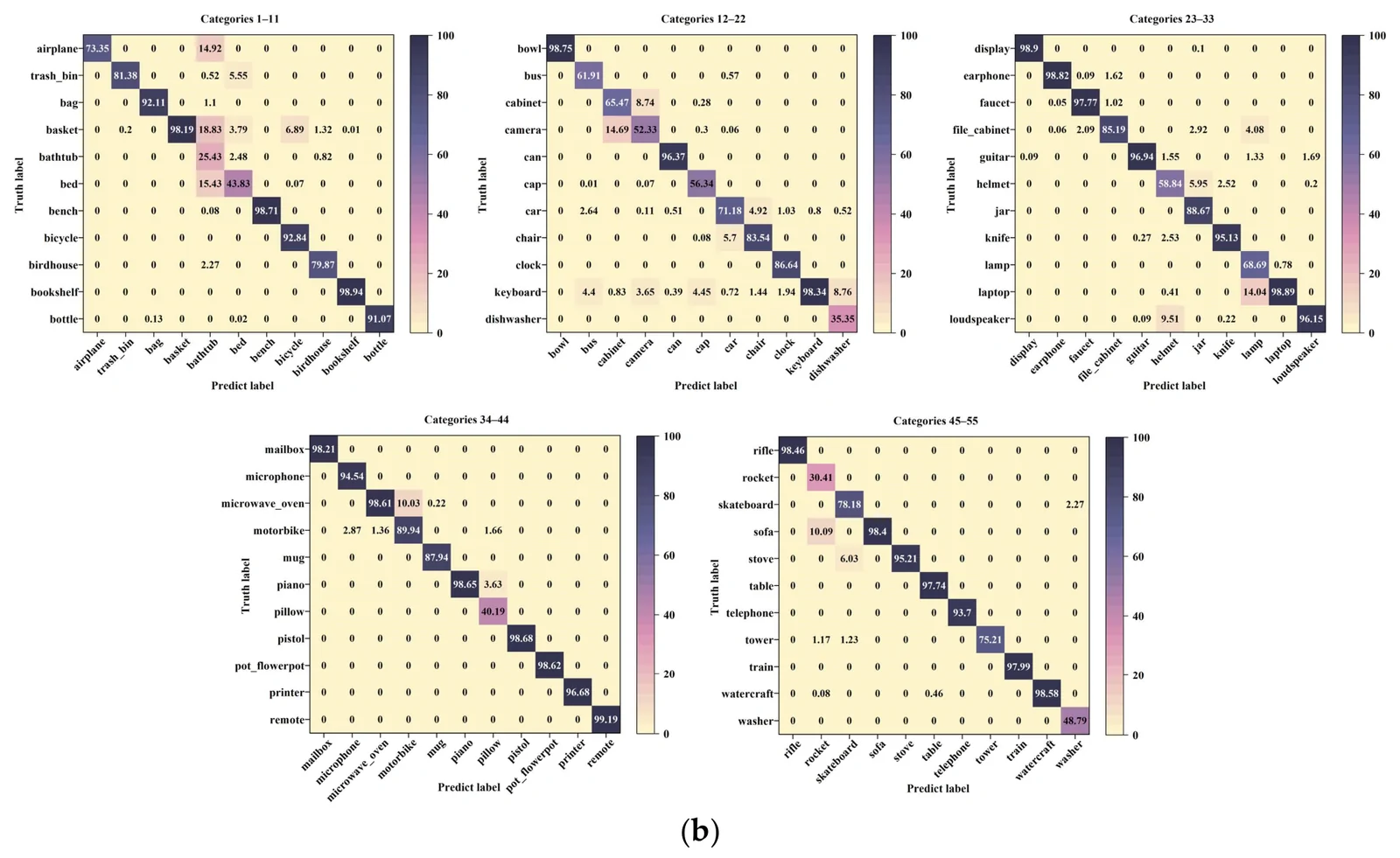
(**a**). Confusion matrix on ModelNet40. (**b**). Confusion matrix on ShapeNet55.

Confusion matrices are used to evaluate per-class classification performance on ModelNet40 and ShapeNet55, as shown in Figure 6a,b.

**Figure 7:**
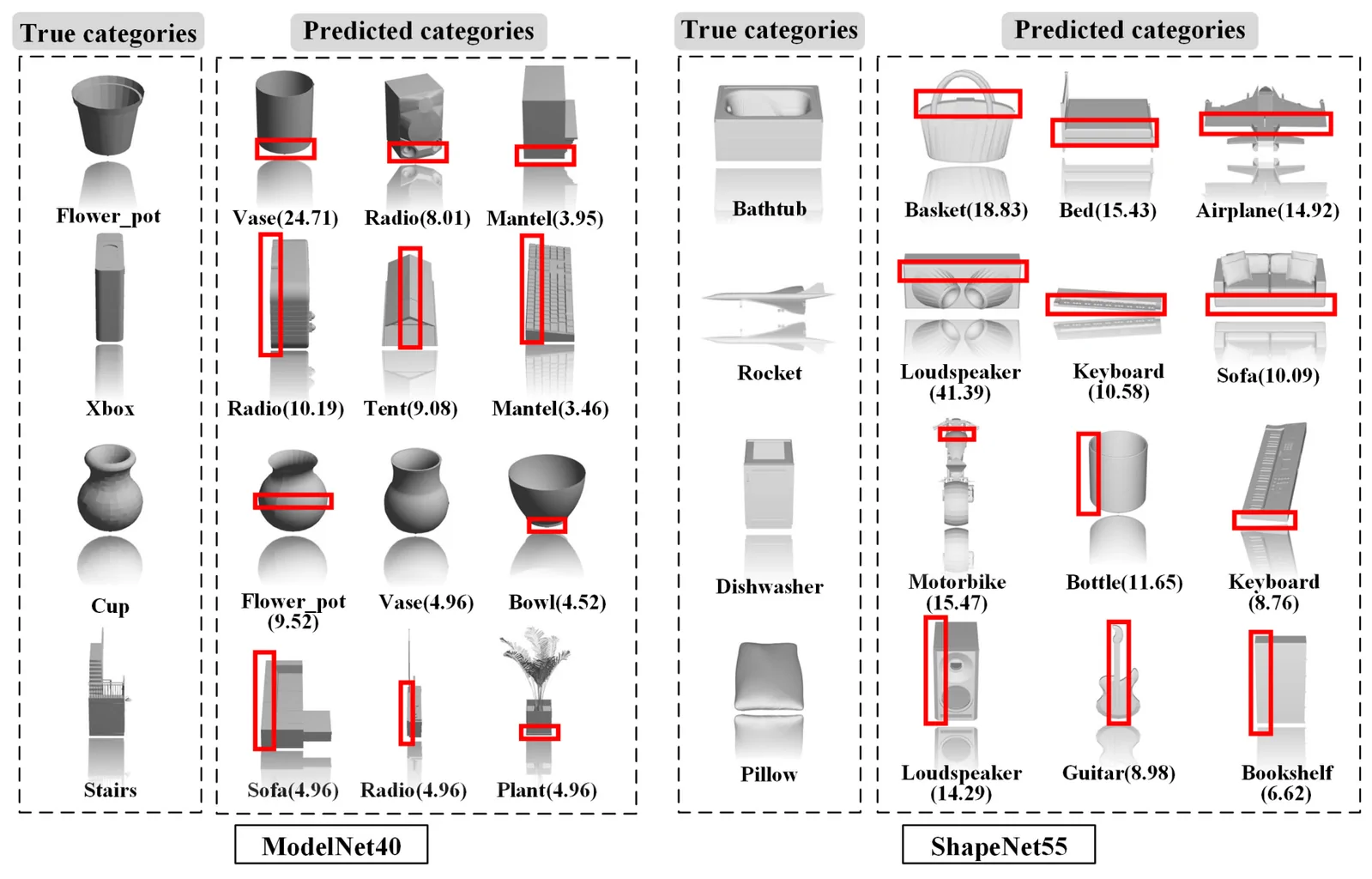
Misclassified category example. Red boxes highlight local structural regions shared by the ground-truth and misclassified categories, which contribute to their geometric or visual confusion.

On ModelNet40, the airplane, bowl, and keyboard classes are classified with 100.00% accuracy, whereas bed, car, and cone achieve 99.10%, 99.65%, and 99.44%, respectively (Figure 6a). These results indicate strong discrimination for categories with distinctive geometry and stable multi-view appearance. Lower accuracies are obtained for flower_pot (63.30%), xbox (74.30%), cup (76.48%), and stairs (79.40%). Flower_pot is most frequently confused with vase, with a confusion rate of 24.71%, primarily because both categories share an open upper structure and a contracted contour, as highlighted in Figure 7. Three broader failure patterns are observed. First, container-like categories, including flower_pot, vase, cup, and bowl, share similar global contours and differ mainly in small local structures. Second, xbox, radio, rocket, and loudspeaker can produce similar elongated or rectangular projections from particular viewpoints, which weakens view-based discrimination. Third, samples with incomplete or atypical local structures, such as bathtub, dishwasher, and pillow, may be assigned to semantically different categories when stable category-specific parts are absent. On ShapeNet55, bookshelf, bench, bowl, display, remote, and pot_flowerpot achieve accuracies of 98.94%, 98.71%, 98.75%, 98.90%, 98.46%, and 96.63%, respectively (Figure 6b). In contrast, bathtub, dishwasher, bed, washer, camera, and chair achieve 18.33%, 35.35%, 43.53%, 48.75%, 52.33%, and 53.54%. SE-CSL therefore remains limited when point-cloud geometry and rendered views provide weak or highly similar category cues. In such cases, global shape dependencies are retained, but fine-grained local differences and adjacent class boundaries are not fully resolved. This limitation is particularly evident for scarce and newly introduced classes because their centers are estimated from fewer training samples.

**Figure 8:**
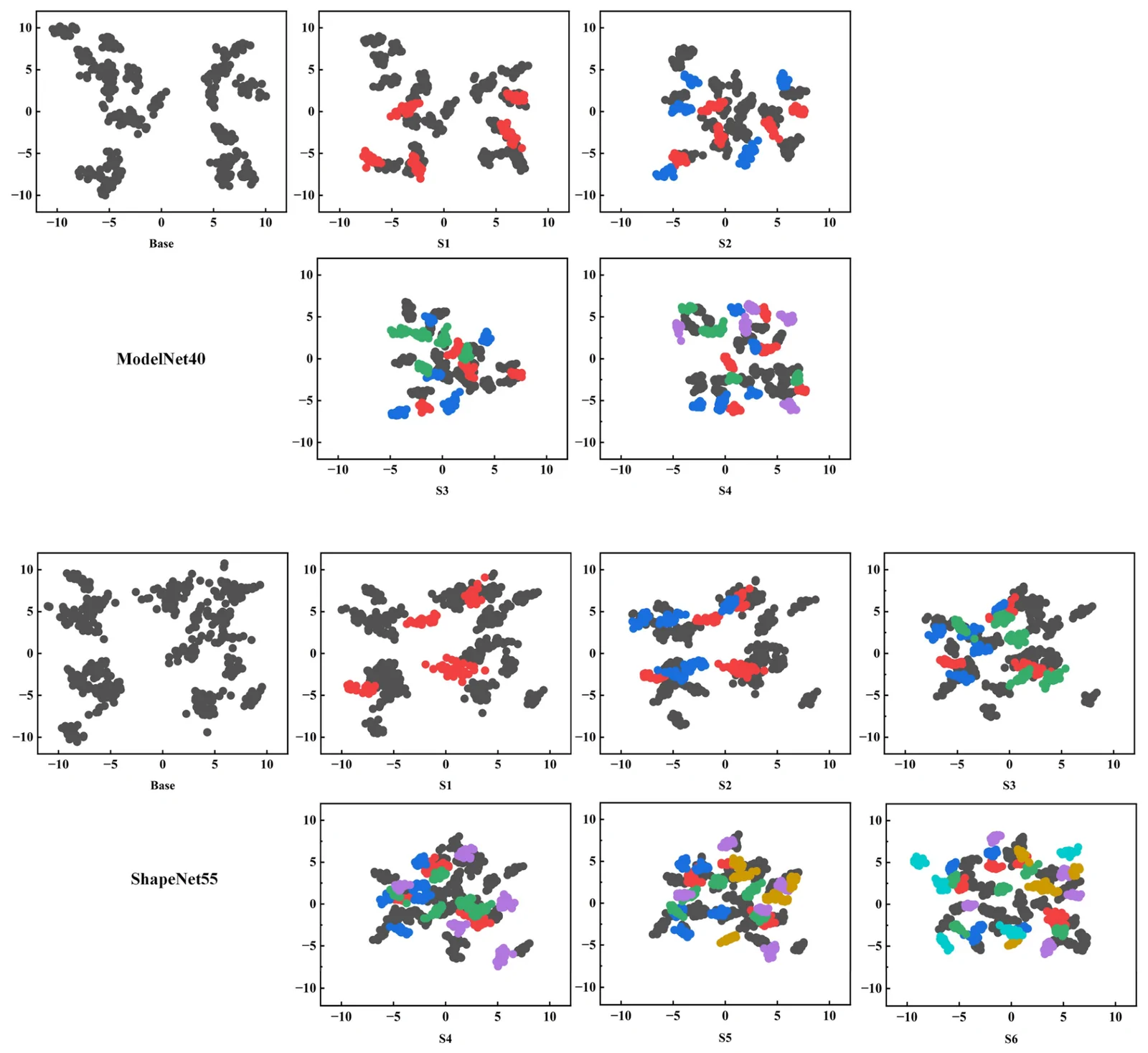
t-SNE visualization of incremental feature distributions. Gray points denote samples from the base phase, while colored points denote samples introduced during successive incremental sessions. Each color represents a ground-truth category.

To assess the discriminative structure of the incremental category space, t-SNE is used to visualize feature distributions on both datasets, as shown in Figure 8.

Each point represents a sample in the latent space and is colored according to its ground-truth class. Gray points correspond to base phase samples, whereas colored points represent samples introduced during incremental sessions. On ModelNet40, the base classes form several distinct clusters, which indicates that the initial category space is discriminative. As new classes are introduced from S1 to S4, their samples form relatively compact clusters within the existing space. Most new-class clusters remain separated from base-class samples, and the structure of the old classes is largely preserved. ShapeNet55 exhibits denser and more locally entangled clusters, particularly from S4 to S6. The results show that the incremental space constructed by the proposed SE-CSL preserves the stability of old-class feature structures during the gradual introduction of new categories and forms relatively independent discriminative regions for new classes, which effectively supports unified recognition of old and new classes under open long-tailed scenarios.

**Figure 9:**
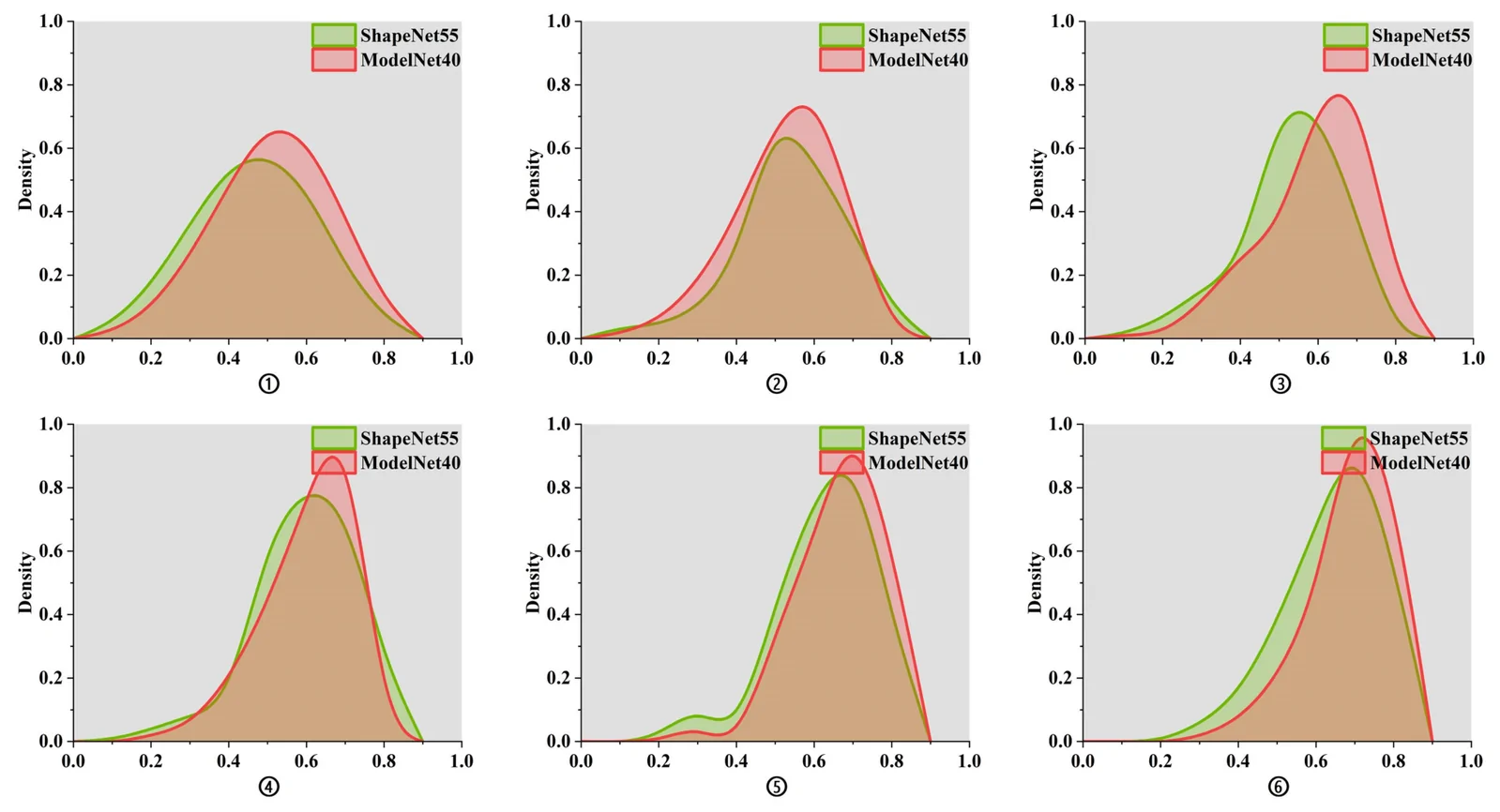
Confidence-score density distributions. Configurations ①–⑥ follow the definitions in Table 11: point-cloud modality only, multi-view modality only, dual-modality input, dual-modality input with Mamba, dual-modality input with Mamba and category-space learning, and the complete SE-CSL model, respectively.

To further examine the incremental learning behavior in category space, Figure 9 presents the density distributions of prediction confidence on ModelNet40 and ShapeNet55. Higher confidence indicates greater certainty in the assigned category.

**Figure 10:**
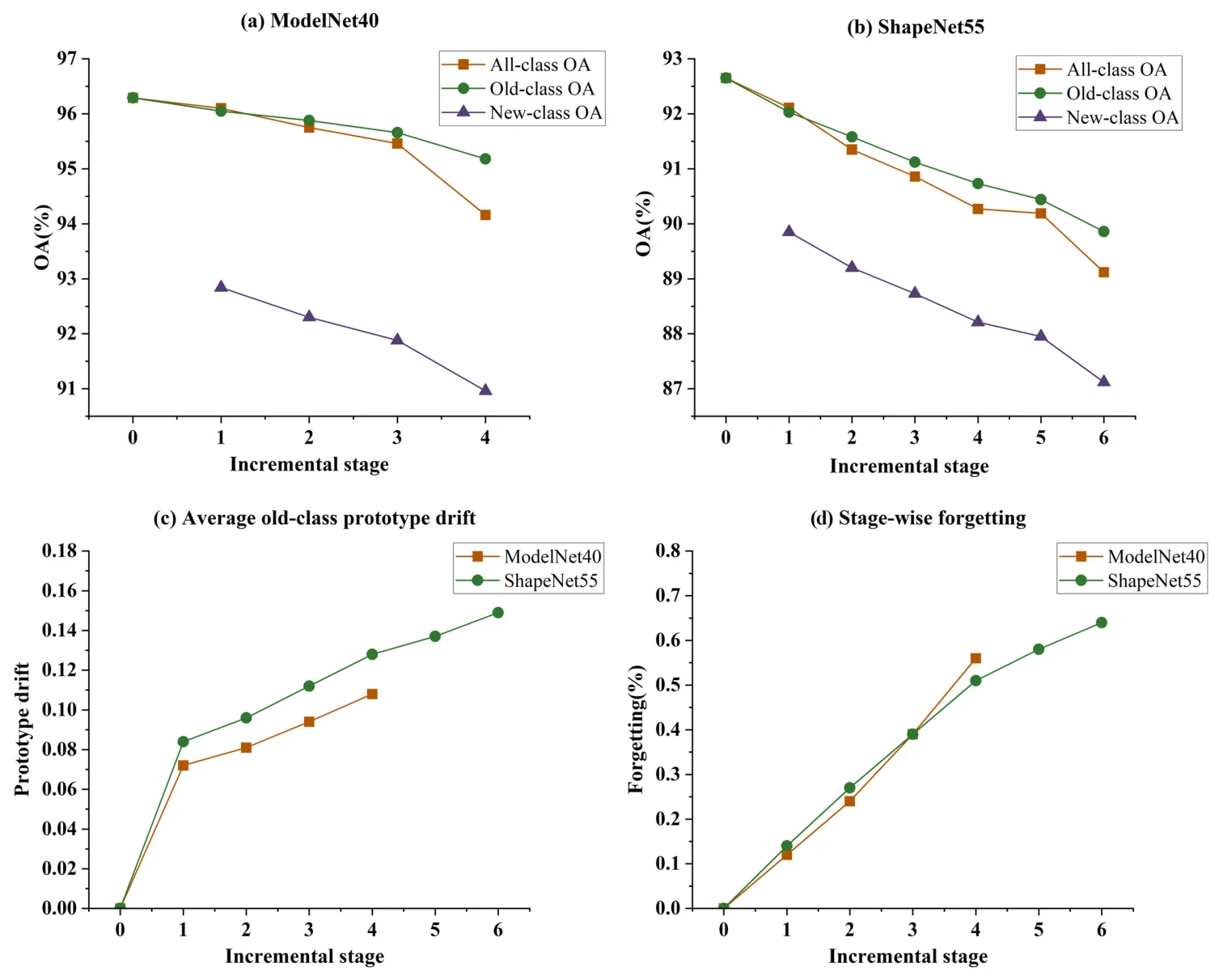
Visualization of incremental learning behavior.

Figure 10 further analyzes the incremental behavior of SE-CSL. Figure 10a,b show that the all-class, old-class, and new-class OA values decrease gradually on ModelNet40 and ShapeNet55, and old-class performance remains relatively stable. Figure 10c shows a slow increase in prototype drift, and Figure 10d reports limited forgetting across stages. These results indicate controlled prototype evolution and effective preservation of previously learned knowledge.

## 5. Conclusions and Discussion

We propose SE-CSL for open long-tailed multimodal 3D model classification. The method constructs a balanced and extensible category space from complementary point-cloud and multi-view representations. Mamba-based dependency modeling integrates heterogeneous features, while category-space learning jointly addresses long-tailed imbalance, scarce-class instability, and ambiguous hard-sample boundaries. Incremental category-space expansion then incorporates new classes and preserves previously learned category information. Experiments on ModelNet40 and ShapeNet55 show that SE-CSL maintains strong classification performance under both class imbalance and incremental category introduction. The learned category space also improves scarce-class representation and retains the discrimination of previously observed classes.

Nevertheless, SE-CSL still has several limitations. Performance decreases for categories with similar global contours and subtle local differences because fine-grained structural cues are not always preserved. Category center estimation can also be unstable for extremely scarce or newly introduced classes, and prototype preservation does not completely prevent cumulative forgetting over long incremental sequences. Future work will investigate finer local representations, more stable center updates, stronger forgetting control, lightweight multimodal deployment, and evaluation on larger open-world 3D datasets.

## Figures and Tables

**Table 1:** Methodological comparison between SE-CSL and representative related methods.

**Method**	**Multimodal 3D Representation**	**Long-Tailed Base Learning**	**Few-Shot New-Class Stabilization**	**Hard-Boundary Optimization**
MM-Point [4]	✓	–	–	–
OpenView [44]	✓	–	–	–
OpenShape [38]	✓	–	–	–
Class-Balanced Loss [11]	–	✓	–	–
Balanced Meta-Softmax [12]	–	✓	–	–
Focal Loss [13]	–	–	–	Partial
Prototypical Networks [14]	–	–	Partial	–
LUCIR [16]	–	–	–	–
SE-CSL	✓	✓	✓	✓
**Method**	**Old-Class** **Preservation**	**Incremental Category** **Expansion**	**Unknown-Class Rejection**	**Unified Category-Space** **Optimization**
MM-Point [4]	–	–	–	–
OpenView [44]	–	–	Partial	–
OpenShape [38]	–	–	Partial	–
Class-Balanced Loss [11]	–	–	–	–
Balanced Meta-Softmax [12]	–	–	–	–
Focal Loss [13]	–	–	–	–
Prototypical Networks [14]	–	–	–	Partial
LUCIR [16]	✓	✓	–	–
SE-CSL	✓	✓	–	✓

Note: ✓ indicates that the corresponding capability is explicitly supported; “Partial” indicates partial support; and “–” indicates that the capability is not addressed.

**Table 2:** Comparison of the proposed protocol with established learning protocols.

Protocol	Base-Class Distribution	New Categories	Supervision for New Categories	Evaluation Scope	Unknown-Class Rejection	Main Objective
[11,12]	Imbalanced	No new classes	Not applicable	Fixed closed-set classes	No	Reduce class frequency bias
[16]	Usually balanced	Sequentially introduced	Regular labeled training samples	All seen classes	No	Preserve old-class knowledge
[17]	Usually balanced	N-way K-shot sessions	K labeled samples per new class	All seen classes	No	Learn new classes from limited samples
[38]	Large-scale training	Unseen semantic categories	Zero-shot	Seen and unseen categories	Partial	Generalize to broader semantic categories
Proposed protocol	Long-tailed head/medium/tail base classes	Fixed 5-way 5-shot sessions	Five labeled samples per new class	All seen classes	No	Joint long-tailed learning and few-shot category-space expansion

**Table 3:** Open long-tailed incremental protocol on ModelNet40 and ShapeNet55.

**Datasets**	**Total Classes**	**Base Classes**	**Incremental Sessions**	**Class Schedule**
ModelNet40	40	20	4	20 + 5 + 5 + 5 + 5
ShapeNet55	55	25	6	25 + 5 + 5 + 5 + 5 + 5 + 5
**Datasets**	**Base H/M/T Split**	**Samples per H/M/T Class**	**Incremental Setting**	**Evaluation Classes**
ModelNet40	8/6/6	80/40/20	5-way 5-shot	All seen classes
ShapeNet55	10/8/7	150/75/30	5-way 5-shot	All seen classes

**Table 4:** Reference comparison with conventional 3D classification methods.

Datasets	Methods	Modal Type	FLOPs (G)	Params (M)	OA
ModelNet40	PEVA-Net [52]	Multi-view	70.5	151.2	84.48
	MV-CLIP [53]	Multi-view	70.4	150	84.44
	ViT-GE [54]	Multi-view	17.60	85.90	93.50
	Pointwise CNN [55]	Point cloud	1.2	2.5	87.07
	Gao-PointNet [56]	Point cloud	2	3.5	88.40
	Chen-M2CP [57]	Multimodal	3	13.2	86.13
	DiffCLIP [58]	Multimodal	16	151	80.60
	Ours	Multi-view + Point cloud	18.9	38.6	94.16
ShapeNet55	PEVA-Net [52]	Multi-view	70.5	151.2	74.65
	MV-CLIP [53]	Multi-view	70.4	150	66.17
	Point-FEMAE (noisy) [59]	Point cloud	5.0	27.4	88.63
	PCP-MAE (noisy) [60]	Point cloud	4.8	22.1	88.24
	Ours	Multi-view + Point cloud	18.9	38.6	89.12

**Table 5:** Comparison with recent specialized incremental and open-world 3D recognition methods (%).

**Datasets**	**Methods**	**Samples per H/M/T Class**	**mAcc**	**OA**
ModelNet40	CMGR [61]	Original distribution	/	65.90
	OpenView [8]	zero-shot	/	87.50
	Ours	80/40/20	91.75	94.16
ShapeNet55	CMGR [61]	Original distribution	NR	86.40
	ILPC [62]	Original distribution	82.27	/
	Ours	150/75/30	83.27	89.12
**Datasets**	**Methods**	**Incremental Sessions**	**Incremental Setting**	**Forgetting ↓**
ModelNet40	CMGR [61]	4	5 classes/session, few-shot	8.7
	OpenView [8]	0	40-class zero-shot recognition	/
	Ours	4	5-way 5-shot	0.56
ShapeNet55	CMGR [61]	6	5 classes/session, few-shot	1.2
	ILPC [62]	6	5 classes/session	/
	Ours	6	5-way 5-shot	0.64

Note: ↓ indicates that a lower value is better. CMGR denotes Cross-Modal Geometric Rectification framework and ILPC denotes Incremental Learning with Part-Concept Awareness framework.

**Table 6:** Comparative experiments on different datasets (%).

**Datasets**	**Recall**	**Precision**	**F1**	**mAcc**	**OA**
ShapeNet55	85.91 ± 0.33	87.10 ± 0.43	86.50 ± 0.35	83.27 ± 0.63	89.12 ± 0.28
ModelNet40	93.09 ± 0.16	93.68 ± 0.22	93.38 ± 0.17	91.75 ± 0.35	94.16 ± 0.19
**Datasets**	**Training Time (h)**	**Inference Time (ms** **)**	**Peak GPU Memory (GB)**		**Scalability (%)**
ShapeNet55	15	35	12		6.5
ModelNet40	12	30	10.5		5.3

**Table 7:** Experimental results of the proposed SE-CSL on different ModelNet40 datasets (%).

Method	Occluded-ModelNet40		Rendered-ModelNet40		ModelNet40	
	mAcc	OA	mAcc	OA	mAcc	OA
ResNet50	66.91 ± 0.26	69.54 ± 0.50	77.24 ± 0.11	82.42 ± 0.19	80.18 ± 0.27	83.07 ± 0.24
DGCNN	70.82 ± 0.27	74.08 ± 0.25	81.30 ± 0.14	85.04 ± 0.21	84.20 ± 0.18	89.61 ± 0.30
Transformer	75.13 ± 0.30	77.04 ± 0.27	82.91 ± 0.24	85.38 ± 0.13	89.26 ± 0.07	91.38 ± 0.22
SE-CSL w/o Mamba	72.87 ± 0.25	76.34 ± 0.09	79.07 ± 0.19	83.72 ± 0.60	86.81 ± 0.12	90.80 ± 0.04
SE-CSL w/o CSL	70.46 ± 0.13	75.95 ± 0.23	81.18 ± 0.24	85.13 ± 0.42	88.00 ± 0.34	91.25 ± 0.31
SE-CSL w/o IL	75.33 ± 0.19	78.80 ± 0.18	83.36 ± 0.05	85.96 ± 0.03	89.43 ± 0.61	92.88 ± 0.26
SE-CSL	77.44 ± 0.50	80.82 ± 0.38	85.60 ± 0.27	88.53 ± 0.23	91.75 ± 0.35	94.16 ± 0.19

**Table 8:** Parameter experiments of the Mamba module (%).

Dim	Kern	ShapeNet55	ModelNet40	ShapeNet55	ModelNet40	ShapeNet55	ModelNet40
		Expan = 1	Expan = 1	Expan = 2	Expan = 2	Expan = 4	Expan = 4
8	2	82.00 ± 0.36	87.09 ± 0.31	85.07 ± 0.34	92.09 ± 0.27	80.27 ± 0.41	89.70 ± 0.16
	4	81.40 ± 0.22	88.05 ± 0.28	85.49 ± 0.28	91.28 ± 0.33	81.66 ± 0.38	90.63 ± 0.30
	6	81.68 ± 0.14	88.70 ± 0.19	86.34 ± 0.31	91.40 ± 0.25	81.40 ± 0.05	90.16 ± 0.29
16	2	84.90 ± 0.34	91.10 ± 0.32	88.40 ± 0.19	92.71 ± 0.42	82.74 ± 0.29	92.63 ± 0.17
	4	85.22 ± 0.02	92.09 ± 0.27	88.38 ± 0.21	93.28 ± 0.30	83.61 ± 0.33	92.11 ± 0.33
	6	85.34 ± 0.32	92.24 ± 0.44	89.12 ± 0.28	94.16 ± 0.19	84.21 ± 0.24	91.80 ± 0.24
32	2	81.07 ± 0.37	85.40 ± 0.31	83.82 ± 0.30	90.14 ± 0.62	79.82 ± 0.40	89.62 ± 0.11
	4	80.55 ± 0.25	84.26 ± 0.27	84.12 ± 0.25	91.83 ± 0.21	80.52 ± 0.36	89.70 ± 0.21
	6	80.29 ± 0.08	86.31 ± 0.20	83.70 ± 0.32	90.55 ± 0.20	81.88 ± 0.17	88.52 ± 0.06

**Table 9:** Parameter experiments of the category-space learning module (%).

Alpha	Proto	ShapeNet55	ModelNet40	ShapeNet55	ModelNet40	ShapeNet55	ModelNet40
		*τ* = 0.35	*τ* = 0.35	*τ* = 0.5	*τ* = 0.5	*τ* = 0.65	*τ* = 0.65
0.3	1	82.16 ± 0.45	88.09 ± 0.33	86.29 ± 0.21	89.90 ± 0.31	83.65 ± 0.45	90.71 ± 0.05
	2	83.22 ± 0.14	89.71 ± 0.40	86.71 ± 0.33	90.13 ± 0.05	84.22 ± 0.23	89.60 ± 0.04
	3	83.91 ± 0.07	88.60 ± 0.09	85.23 ± 0.50	90.80 ± 0.29	84.17 ± 0.37	88.57 ± 0.60
0.6	1	87.36 ± 0.32	92.46 ± 0.28	89.00 ± 0.17	93.25 ± 0.14	86.23 ± 0.02	91.55 ± 0.23
	2	87.25 ± 0.35	91.83 ± 0.70	89.12 ± 0.28	94.16 ± 0.19	86.40 ± 0.16	92.60 ± 0.19
	3	86.11 ± 0.18	92.36 ± 0.22	88.19 ± 0.11	93.06 ± 0.26	87.18 ± 0.33	91.28 ± 0.40
0.9	1	85.90 ± 0.40	90.25 ± 0.34	87.05 ± 0.38	91.45 ± 0.18	85.26 ± 0.35	90.61 ± 0.30
	2	85.33 ± 0.32	90.30 ± 0.07	87.40 ± 0.51	92.33 ± 0.14	83.72 ± 0.18	90.08 ± 0.29
	3	84.17 ± 0.18	91.45 ± 0.32	87.23 ± 0.27	92.60 ± 0.25	84.33 ± 0.16	89.12 ± 0.24

**Note:** Alpha denotes the long-tail balancing parameter; Proto denotes the global few-shot stabilization factor; and denotes the hard-sample selection threshold.

**Table 10:** Long-tailed class-incremental learning results (%).

Dataset	Total Classes	Base Classes	Phase	Seen Classes	New Classes	mAcc	OA
ModelNet40	40	20	Base	20	0	95.52 ± 0.15	96.29 ± 0.56
			S1	25	5	93.11 ± 0.19	96.10 ± 0.13
			S2	30	5	92.82 ± 0.34	95.75 ± 0.31
			S3	35	5	92.30 ± 0.28	95.46 ± 0.27
			S4	40	5	91.75 ± 0.35	94.16 ± 0.19
ShapeNet55	55	25	Base	25	0	88.52 ± 0.51	92.65 ± 0.63
			S1	30	5	87.63 ± 0.31	92.11 ± 0.17
			S2	35	5	87.35 ± 0.29	91.35 ± 0.38
			S3	40	5	85.86 ± 0.16	90.86 ± 0.42
			S4	45	5	84.33 ± 0.55	90.27 ± 0.64
			S5	50	5	84.20 ± 0.21	90.19 ± 0.33
			S6	55	5	83.27 ± 0.63	89.12 ± 0.28

**Table 11:** Ablation experiment (%).

**Method**						**ShapeNet55**		**ModelNet40**	
	**(a)**	**(b)**	**(c)**	**(d)**	**(e)**	**mAcc**	**OA**	**mAcc**	**OA**
①	√	×	×	×	×	70.02 ± 0.09	75.63 ± 0.81	79.60 ± 0.31	82.13 ± 0.58
②	×	√	×	×	×	71.68 ± 0.15	77.90 ± 0.07	82.61 ± 0.44	86.06 ± 0.25
③	√	√	×	×	×	79.36 ± 0.47	82.54 ± 0.40	86.30 ± 0.08	89.66 ± 0.41
④	√	√	√	×	×	81.00 ± 0.25	86.30 ± 0.24	88.25 ± 0.63	91.90 ± 0.70
⑤	√	√	√	√	×	81.59 ± 0.35	87.01 ± 0.30	89.43 ± 0.61	92.88 ± 0.26
⑥	√	√	√	√	√	83.27 ± 0.63	89.12 ± 0.28	91.75 ± 0.35	94.16 ± 0.19
**Method**						**Occluded-ModelNet40**		**Rendered-ModelNet40**	
	**(a)**	**(b)**	**(c)**	**(d)**	**(e)**	**mAcc**	**OA**	**mAcc**	**OA**
①	√	×	×	×	×	66.23 ± 0.35	69.85 ± 0.54	72.63 ± 0.10	76.94 ± 0.26
②	×	√	×	×	×	69.30 ± 0.50	72.51 ± 0.32	74.25 ± 0.63	79.30 ± 0.55
③	√	√	×	×	×	71.83 ± 0.43	75.62 ± 0.04	79.80 ± 0.40	82.34 ± 0.30
④	√	√	√	×	×	73.60 ± 0.21	78.12 ± 0.35	81.33 ± 0.25	85.40 ± 0.70
⑤	√	√	√	√	×	75.33 ± 0.19	78.80 ± 0.18	83.36 ± 0.05	85.96 ± 0.03
⑥	√	√	√	√	√	77.44 ± 0.50	80.82 ± 0.38	85.60 ± 0.27	88.53 ± 0.23

Note: √ and × indicate that the corresponding component is included and excluded, respectively.

**Table 12:** Ablation study of category-space learning losses (%).

*L*_long_	*L*_few_	*L*_hard_	ShapeNet55		ModelNet40	
			mAcc	OA	mAcc	OA
×	×	×	78.64 ± 0.51	85.76 ± 0.12	87.00 ± 0.34	91.25 ± 0.31
√	×	×	81.76 ± 0.11	87.22 ± 0.30	88.75 ± 0.38	92.33 ± 0.28
×	√	×	80.18 ± 0.52	86.20 ± 0.34	89.41 ± 0.16	92.06 ± 0.25
×	×	√	81.63 ± 0.06	87.15 ± 0.40	89.00 ± 0.44	91.74 ± 0.30
√	√	√	83.27 ± 0.63	89.12 ± 0.28	91.75 ± 0.35	94.16 ± 0.19

## Data Availability

The data used in the study are openly available in ModelNet40 at https://modelnet.cs.princeton.edu/ (accessed on 24 July 2026) and ShapeNet55 at https://shapenet.org/ (accessed on 24 July 2026).
